# Community Knowledge and Attitudes and Health Workers' Practices regarding Non-malaria Febrile Illnesses in Eastern Tanzania

**DOI:** 10.1371/journal.pntd.0002896

**Published:** 2014-05-22

**Authors:** Beatrice Chipwaza, Joseph P. Mugasa, Iddy Mayumana, Mbaraka Amuri, Christina Makungu, Paul S. Gwakisa

**Affiliations:** 1 Nelson Mandela African Institute of Science and Technology, Arusha, Tanzania; 2 Ifakara Health Institute, Ifakara, Tanzania; 3 National Institute for Medical Research, Amani Medical Research Centre, Muheza, Tanga, Tanzania; 4 Jhpiego, Dar-es-Salaam, Tanzania; University of Ghana, Ghana

## Abstract

**Introduction:**

Although malaria has been the leading cause of fever for many years, with improved control regimes malaria transmission, morbidity and mortality have decreased. Recent studies have increasingly demonstrated the importance of non-malaria fevers, which have significantly improved our understanding of etiologies of febrile illnesses. A number of non-malaria febrile illnesses including Rift Valley Fever, dengue fever, Chikungunya virus infection, leptospirosis, tick-borne relapsing fever and Q-fever have been reported in Tanzania. This study aimed at assessing the awareness of communities and practices of health workers on non-malaria febrile illnesses.

**Methods:**

Twelve focus group discussions with members of communities and 14 in-depth interviews with health workers were conducted in Kilosa district, Tanzania. Transcripts were coded into different groups using MaxQDA software and analyzed through thematic content analysis.

**Results:**

The study revealed that the awareness of the study participants on non-malaria febrile illnesses was low and many community members believed that most instances of fever are due to malaria. In addition, the majority had inappropriate beliefs about the possible causes of fever. In most cases, non-malaria febrile illnesses were considered following a negative Malaria Rapid Diagnostic Test (mRDT) result or persistent fevers after completion of anti-malaria dosage. Therefore, in the absence of mRDTs, there is over diagnosis of malaria and under diagnosis of non-malaria illnesses. Shortages of diagnostic facilities for febrile illnesses including mRDTs were repeatedly reported as a major barrier to proper diagnosis and treatment of febrile patients.

**Conclusion:**

Our results emphasize the need for creating community awareness on other causes of fever apart from malaria. Based on our study, appropriate treatment of febrile patients will require inputs geared towards strengthening of diagnostic facilities, drugs availability and optimal staffing of health facilities.

## Author Summary

Understanding the awareness of the community on non-malaria febrile illnesses is crucial, especially during the recent decline of malaria episodes of malaria. This study conducted focus group discussions with communities to assess their awareness of non-malaria febrile illnesses. In addition, in-depth interviews with health workers were conducted to explore their views and practices related to diagnosis and management of these illnesses. We identified that the awareness of the study participants on non-malaria febrile illnesses was low and the majority believed that most instances of fever are due to malaria. Moreover, the participants could not mention the right causes of fever and many had inappropriate beliefs about possible causes of fever. Health workers from our study looked for non-malaria febrile illnesses when a febrile patient had negative mRDT result or there was persistence of fever following completion of anti-malarial dosage. Shortages of diagnostic facilities were identified as one of the impediments to proper diagnosis of febrile illnesses. These findings indicate the need for creation of public awareness on causes of fever other than malaria. We recommend appropriate measures be taken by the government and other stake holders to improve health care services delivery particularly at primary health care facilities.

## Introduction

Febrile illnesses due to different etiological agents are the common causes of morbidity and mortality in developing countries [1]. Malaria has been the leading cause of fever in sub- Saharan Africa for many years [2]. For instance, in Tanzania, malaria was contributing to about 42% of hospital diagnoses and 32% of hospital deaths in the last decade [3]. Accordingly, presumptive treatment of all febrile illnesses in children under five years with anti-malarial drugs was adopted as policy in many countries of sub-Saharan Africa [4]. However, in recent years, there has been gain in malaria control strategies which has led to decreased malaria prevalence particularly in endemic countries [5]–[7]. The decrease in malaria burden has also been indicated by the 2012 World Malaria Report where there is a good achievement in worldwide reduction of malaria transmission, morbidity and mortality [8]. The decline in malaria transmission is mainly a result of increased coverage of different malaria control strategies that have been implemented for several years. This includes the use of long lasting insecticide treated bed nets, indoor residual spraying, intermittent presumptive treatment of malaria during pregnancy or intermittent presumptive treatment of malaria to infants and treatment with effective anti-malaria drugs such as Artemisinin-based Combination Therapies [5], [9]. The decrease in malaria transmission led World Health Organization (WHO) to change its policy in 2010 where anti-malarial treatment is initiated after parasitological confirmation [10]. However, the decline in trend of malaria transmission in many malaria-endemic countries corresponds to an increasing proportion of febrile patients who are diagnosed as not having malaria [11], [12]. Recent studies have increasingly demonstrated the importance of non-malaria fevers, which have significantly improved our understanding of etiologies of febrile illnesses [13], [14]. In this regard, a reasonable proportion of febrile illnesses are now ascribed to be non-malaria febrile illnesses [13] and episodes of such diseases are reported to increase [12]. In Tanzania, diseases such as respiratory tract infections, urinary tract infections, typhoid fever and rotavirus infection are among non-malaria febrile illnesses that have been commonly affecting people particularly children [15]–[18]. A study conducted in Dar es Salaam and Ifakara had shown that among 1005 children, 498 (50%) had acute respiratory infection, while 54 (5.4%) had urinary tract infections and 33 (3.3) had typhoid fever [18]. Diseases such as Rift Valley Fever (RVF), dengue fever, Chikungunya virus infection, leptospirosis, tick-borne relapsing fever, Q-fever, rotavirus infection and brucellosis have also been reported in Tanzania [13], [14], [19]–[24]. A recent study conducted in northern Tanzania has reported the occurrence of 55 (7.9%) cases of Chikungunya virus infection, 40 (33.9%) cases of leptospirosis, 24 (20.3%) cases of Q-fever and 16 (13.6%) cases of brucellosis among 870 admitted febrile patients [13]. Some non-malaria febrile illnesses may contribute to high morbidity and mortality in humans. For instance, rotavirus takes the lives of more than 8,100 Tanzanian children under five each year [25]. Furthermore, the most recent outbreak of RVF in 2006/2007 which occurred in 10 regions of Tanzania mainland [26] and in other countries such as Kenya and Somalia were associated with widespread morbidity and mortality in humans [27].

The diagnosis of non-malaria febrile illnesses poses a challenge since many of these illnesses may have similar symptoms with malaria and thus making their clinical diagnosis difficult [28]. Also, non-malaria febrile illnesses could have common overlapping manifestations and therefore, this absence of specific symptoms make it difficult to distinguish several non-malaria febrile conditions that often occur in the same area [29]. Clinical overlap between diseases may result in inappropriate antimicrobial therapy and therefore, laboratory tests for differential diagnosis of causative agent are essential.

Following a long tradition of regarding malaria as the leading cause of fever, it is important for the community to understand the other causes of fever apart from malaria particularly during this period when the episodes of malaria related fevers are reported to decrease [8], [11], [30]. Understanding the awareness of the community on non-malaria febrile illnesses is critical and relevant particularly in management and control of such illnesses. Despite their importance, only few studies aiming at assessing the awareness of the communities regarding non-malaria febrile illnesses have been conducted in Tanzania [31], [32]. Therefore, this study intended to contribute in filling the information gap by assessing the knowledge and attitude of the communities regarding non-malaria febrile illnesses. In addition, the study explored treatment seeking behaviors for febrile illnesses among community members. Following the longstanding practice of treating most fevers as malaria, health workers may still treat febrile patients with anti-malarial drugs even if the patients had a negative test results. Studies from Tanzania and other countries like Zambia, Uganda and Burkina Faso have indicated that febrile patients were prescribed anti-malarial drugs following negative mRDT/microscopy result [33]–[36]. Therefore, there is a need to know the management of febrile patients following the decline in the incidence of malaria. The current study also assessed health workers' practices related to diagnosis and treatment of febrile patients.

## Methods

### Study area

The study was conducted in Kilosa district which is one of the six districts in Morogoro region, located in eastern Tanzania. The district borders with Tanga and Manyara regions to the north and Mvomero district and Mikumi National Park to the east. On the western border are Dodoma and Iringa regions whereas to the south it borders with Kilombero district. The district lies between latitudes 6° south and 8° south and longitudes 36°30′ east and 38° west. The area has semi humid climate with an average rainfall of 800 mm annually. The early rains start in November and end in January followed by heavy rainfall between March and May. The district experiences a long dry season from June to October and the average annual temperature is 24.6°C. The district has an area of 14,245 square kilometers and has a population of 438,175 people [37]. It consists of a mixture of different ethnic groups predominantly Kaguru, Sagara and Vidunda. The main economic activities are crop production and livestock keeping. More than 77% of people are subsistence farmers and major crops cultivated include maize, cassava, rice, paddy and sorghum whereas the major cash crops are sisal, sugarcane, cotton and oilseeds.

**Figure 1:**
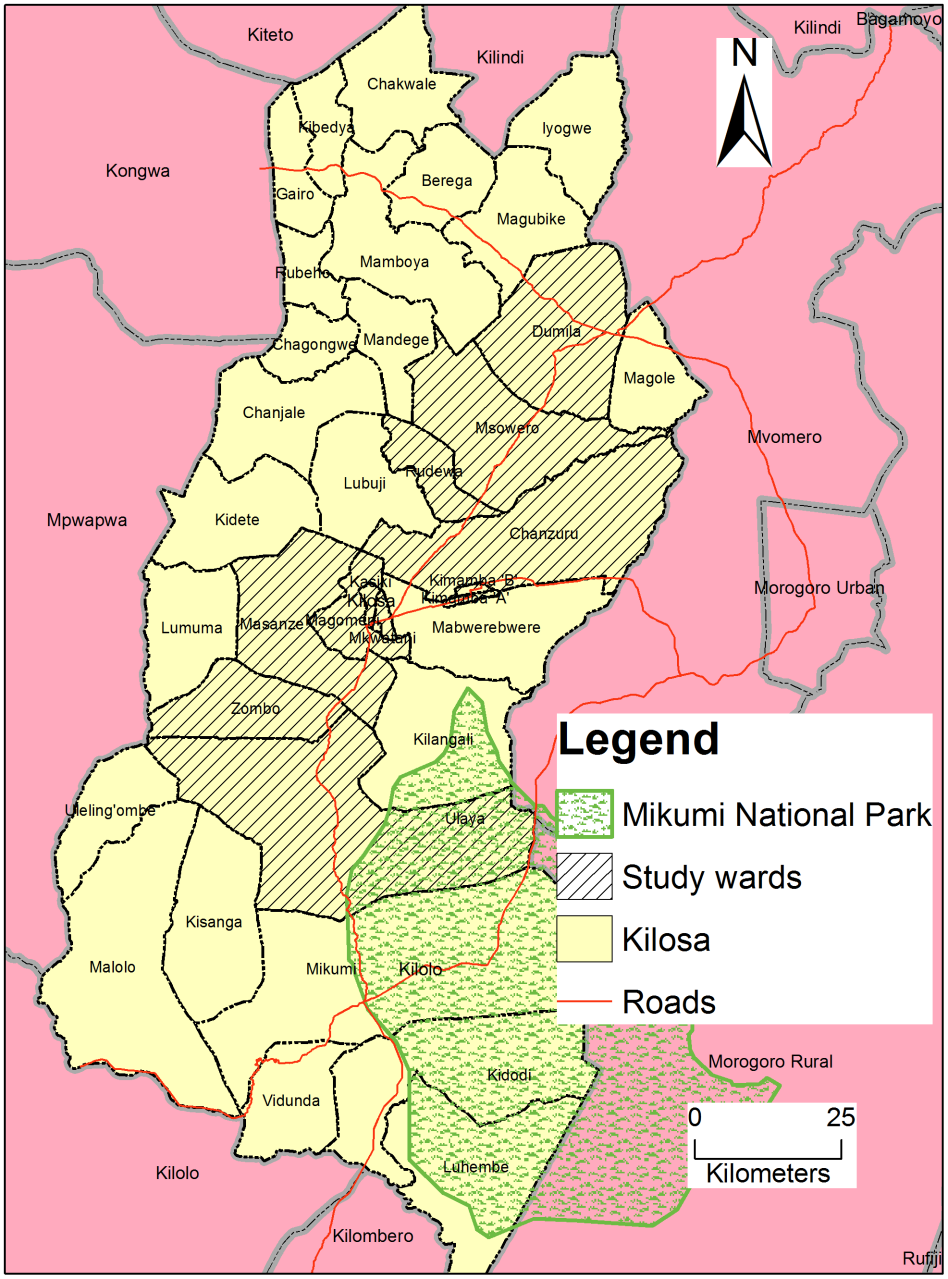
Map of Kilosa district showing the study wards.

Kilosa was selected due to its possession of intensive human activities with livestock as well as its proximity to wildlife from the Mikumi National Park (figure 1), what was expected to be a good interface for zoonotic diseases such as RVF and Brucellosis [38]. Administratively, Kilosa district is divided into 9 divisions, 37 wards and 164 villages [39]. In terms of health care services, Kilosa district has 71 health facilities and among these, there are 3 hospitals, 7 health centers and 61 dispensaries [40]. However, the number of villages exceeds the number of health facilities and hence most health facilities serve more than one village. Kilosa district is an area with holoendemic malaria transmission with seasonal peaks following the long and short rainy seasons [41]. According to Tanzania HIV and Malaria Indicator Survey, in 2007–2008 malaria prevalence was estimated to be 15.7% in Morogoro region [42] and decreased to 13% in the year 2011–2012 [43]. The common non-malaria febrile illnesses that have been reported in Kilosa district include acute respiratory diseases, UTIs and typhoid fever [24]. Data from a platform for health monitoring and evaluation in Tanzania (Sentinel Panel of Districts) have shown that in the year 2011, acute respiratory diseases and UTIs comprised of 20% and 2.5% respectively of total recorded illnesses (77,862) in outpatient department in children aged less than 5 years [44].

This study was specifically conducted in 6 divisions, namely Kimamba, Kilosa town, Magole, Masanze, Rudewa and Ulaya. Within these divisions, 12 wards namely Dumila, Chanzuru, Magomeni, Kilosa, Msowero, Zombo, Ulaya, Kimamba, Mkwatani, Kasiki, Masanze and Rudewa were purposively selected based on (i) geographical representation within the district e.g. Zombo is in the south western part of the district, whereas Dumila ward is in north-eastern part (figure 1), (ii) the presence of government health facilities (iii) connectivity of the wards and ease of accessibility by road.

### Study design

We conducted a cross-sectional study in which qualitative data collection methods were used. Focus group discussions (FGDs) with members of the communities were conducted to assess their knowledge, attitude and perception of community members on non-malarial febrile illnesses. In-depth interviews (IDIs) with health workers were conducted in order to obtain their views about practices related to diagnosis and management of non-malarial febrile illnesses.

### Participants and sampling procedures

Parents, guardians or caregivers aged between 18–59 years for children of less than 10 years were eligible to participate in FGDs. This group of participants was targeted because febrile illnesses have been shown to be common in children and contribute to high proportion of hospital admissions globally, with significant morbidity and mortality [13], [18], [45]. The participants were recruited from different hamlets within the study wards with the assistance of local government and villages leaders. In total 12 FGDs were conducted in urban, peri-urban and rural areas in the selected wards of which 5 FGDs were with men and 7 involved women. Each FGD comprised 6–8 people, but women and men were separately interviewed to give the participants freedom to talk during the discussions. Two FGDs were conducted per day and each FGD took about 60 to 90 minutes. In each of Dumila, Rudewa, Chanzuru and Ulaya wards 2 FGDs were separately conducted for men and women. In Masanze and Zombo wards 2 FGDs were conducted with women and the remaining 2 FGDs (1with men and 1with women) involved participants selected from Kilosa, Kasiki and Magomeni wards.

For IDIs, only health workers who were on duty and attended patients (prescribers) in the health facilities during the study period were eligible to participate into the study. Health workers from 12 health facilities located in Dumila, Chanzuru, Ulaya, Zombo, Kilosa, Magomeni, Msowero, Kimamba, and Mkwatani wards were interviewed. Two health workers were interviewed from each health facility and only one health worker was interviewed at a time. In-depth interviews with health workers on average lasted for one hour and 2 IDIs were conducted per day.

### Data collection

Focus group discussions and IDIs were conducted by a skilled and experienced social scientist who was assisted by an observer and note taker. All discussions and interviews were conducted based on a prepared semi-structured interview guide that consisted of questions corresponding to the research topic. To ensure accuracy of the information, the data collection tool was translated from English to Kiswahili and then back-translated. The interview guide for FGDs consisted of questions about their knowledge on non-malaria febrile illnesses, health care seeking behaviors and their recommendations on non-malaria febrile illnesses (supporting information S1). Health workers were asked questions on the awareness of the communities on non-malaria febrile illnesses, communities' health seeking behaviors, how they perform diagnosis and treatment of febrile patients and their recommendations on proper management of non-malaria febrile illnesses (supporting information S1). During the interviews and discussions, notes were taken and conversations were digitally recorded. Field notes were expanded on the same day of the interview/discussion. All FGDs and IDIs were held in Swahili language which is the most widely spoken language by the community (national language).

### Data management and analysis

FGDs and interviews were transcribed verbatim and translated from Swahili to English. Thereafter, the transcripts were converted into rich text format and imported into MaxQDA, a software for qualitative data analysis [46]. Text files were independently reviewed by the two researchers (IM and CM) before agreeing on the different themes and categories. In case of differing interpretations, the discussion between the researchers took place until the final agreement was reached. The findings were also validated by the interviewing researcher (BC). The agreed themes and categories were then coded. The retrieved segments were analyzed using thematic content analysis and their respective codes were exported to Excel for quantitative analysis.

### Ethics statement

Ethical approval was obtained from Institutional Review Board of Ifakara Health Institute (IHI/IRB/No: 01-2013) and Medical Research Coordinating Committee of Tanzania's National Institute for Medical Research (NIMR/HQ/R.8a/Vol.1X/1472). A written informed consent was obtained from each respondent and participant prior to IDIs or FGDs. To protect identification of the respondents and FGD participants, all personal information that could identify the study participants were only used during the analysis and omitted from the final reports. The participants were assured of anonymity in the presentation and publishing of the data.

## Results

In total, 93 participants were interviewed during FGDs, of which 56 were women and 37 were men. Most of the study participants 87 (93.5%) were subsistence farmers and the majority 73(78.5%) had primary level of education. The demographic characteristics of the study population are shown in Table 1. A total of 12 health facilities were visited, among which were 1 hospital, 2 health centers and 9 dispensaries. Fourteen health workers were interviewed, including 8 clinical officers, 1 assistant clinical officer, 3 nurses and 2 medical attendants. Among 14 health workers, 6 were males and 8 were females. Eleven health workers (11/14) had work experience ranging from 1–20 years whereas 3 health workers (3/14) had work experience of 21–40 years. The study identified five major themes as follows:

**Table 1:** Demographic characteristics of FGD participants.

Category	Subcategory	n	%
Sex	Male	37	39.8
	Female	56	60.2
Age	18–30 y	41	44.1
	31–60 y	52	55.9
Occupation	Subsistence farming	87	93.5
	Business	1	1.1
	Employed	5	5.4
Education level	Illiterate	11	11.8
	Primary	73	78.5
	Secondary	7	7.5
	Tertiary	2	2.2

1. Participants' understanding of fever.
2. Awareness of the community on non-malaria febrile illnesses.
3. Treatment seeking behaviors for febrile illnesses.
4. Health workers' practices towards non malaria febrile illnesses.
5. Capacity for diagnosis and management of febrile illnesses in health facilities.

### Theme 1: Participants' understanding of fever

The study participants were asked to explain what they know about the term “fever” (“homa” in Swahili language). There were different levels of understanding among the participants. The responses provided by majority of the participants did not associate fever with high body temperature. They described fever as an illness condition such as malaria, colic, rheumatism and sleeping sickness or associated it with symptoms such as headache, coughing, rashes and body pain. Others reported that they did not know the exact meaning of the term fever. There were few participants who described fever as a raise of body temperature (hot body).

*“I personally, don't know what fever is.”(FGD-women)*

*“Cold all over the body, severe headache, joint pain; to me, that is fever.”(FGD-women)*

When the participants were asked to mention the causes of fever in children, things such as change of weather (cold, high temperature) and sunlight were listed by many participants. The participants believed that exposure to a cold environment or prolonged stay under the sun by itself can lead to fever. Only a few participants mentioned the right cause of fever which included illnesses like measles, tuberculosis (TB), typhoid fever and UTIs and other participants associated fever with symptoms/clinical signs such as flu, coughing and diarrhea.

*“The change of weather causes fever, such as when it's cold, windy or sunny.” (FGD-men)*

*“I don't know whether fever is caused by heat or sunlight. So often you may offer drugs to your children and they could finish their dosages, but the fever does not disappear.” (FGD-women)*

*“TB and flu are the main causes of fever.” (FGD women)*

Moreover, inappropriate beliefs were perceived as causes of fever by the participants. For example, the presence of false teeth (*meno ya plastiki in Swahili language*), breastfeeding after long-term sunlight exposure of the mother as well as cessation of pulsation in the fontanel were mentioned by some participants.

*“Our wives spend most of the time under the sun. The sunlight renders infection into the breast milk; hence children get fever.”(FGD-men)*

*“When there is cessation of pulsation in the fontanel, it causes pain and this will definitely lead to fever.” (FGD-women)*

### Theme 2: Awareness of the community on non-malaria febrile illnesses

When the FGD participants were asked to mention the causes of fever other than malaria, several of them listed diseases or symptoms which were neither associated with fever nor non-malaria febrile illnesses. The commonly reported diseases/symptoms were headache, colic, hernia and abdominal pain. However, some participants admitted to be unaware of such illnesses. Only a small number of the participants mentioned the correct non-malaria febrile illnesses such as typhoid fever, UTIs (dirty urine), pneumonia, measles and tuberculosis (TB) and sleeping sickness. Both men and women participants had similar level of knowledge, but participants older than 30 years were more knowledgeable than those who were younger. Moreover, the participants from FGDs conducted in rural areas had limited knowledge in comparison with participants from urban and semi-urban areas.

*“A headache can cause fever.” (FGD-women)*

*“Frankly we don't know, unless you explain to us.” (FGD-women)*

*“Hernia.” (FGD-men),*

*“Others include colic, this can cause fever in a child.”(FGD-women)*

It was also revealed that despite the decrease of malaria, the participants believed that most instances of fever were due to malaria. This was noted when several participants mentioned only malaria as the cause of fever. The participants explained that when their children get fever, they mostly associated it with malaria. This was also acknowledged when the participants were asked to describe the meaning of the term fever (theme 1). A considerable number of the participants explained fever is malaria:

*“To be honest, I don't know other diseases. We are aware of malaria. When a child gets sick, we know that it is malaria.”(FGD-women)*

*“As we said earlier, we don't know other causes of fever apart from malaria, I don't know.” (FGD-women)*

During the interviews, health workers were asked to give their views about the knowledge of the community members on non-malaria febrile illnesses. All health workers reported that majority of community members were not aware or had little knowledge on these illnesses. Health workers explained that fevers were perceived to be caused by malaria by several members of communities. This situation was reported to be a challenge to health workers particularly when they want to obtain a comprehensive history of illness from patients since patients explain to health workers that they are suffering from malaria. Health workers identified this wrong perception as an impediment to proper management of patients and emphasized the need for change of attitude.

*“In short they are not aware of other causes of fever. What they know is that fever is synonymous with malaria. They believe that fever is caused by malaria alone.” (IDI-clinical officer)*

*“Parents don't have knowledge on non-malaria fevers. What they think is that, all children with fever have malaria, mRDT results may be negative, but their parents will not believe that they had no malaria.”(IDI-clinical officer)*

*“They don't have sufficient knowledge because once a person is ill or has fever, they think of malaria. People are still ignorant.” (IDI-registered nurse)*

In our study the most common reason for unawareness of the community on non-malaria febrile illnesses reported by the majority of health workers was lack of health education. Health workers pointed out that health education is offered at health facilities but it has not reached a wider community. Several community members live in remote villages and they rarely visit health centres for health care. Health workers emphasized that health education on diseases associated with non malaria fevers will help to create awareness to members of the community.

*“Health education doesn't reach the majority of people since it is only offered here at the health centre, and others live in remote villages.” (IDI-registered nurse)*

### Theme 3: Treatment seeking behaviors for febrile illnesses

Findings from the FGDs revealed that community members from the study population sought treatment from both the health facilities and traditional healers. When queried on their health seeking behavior, the majority of the study participants reported sending their febrile children to the nearby health facility. However, when asked for any alternative treatment, almost half of the participants reported to seek treatment from the traditional healers. Therefore, this indicates that health facilities and traditional healers were both utilized by the participants. Even though several participants reported seeking treatment from health facilities, but interviewed health workers explained that community members rarely visit health facilities. They said the majority of community members live in remote areas and thus long distances pose as an impediment for visiting health facilities. In addition, health workers stated that the habit of seeking treatment from traditional healers was practiced by some members of the community. They further pointed out that sometimes children were sent to health facilities when they were terminally ill. Likewise, some parents/guardians admitted to health workers that delays to send their children to health facilities was due to prior consultations made from traditional healers.

*“It is not true that all go to the health facility as my colleague has put it. Some don't have even the idea of going to a dispensary, what comes into their mind is a traditional healer.”(FGD-women)*

*‘Some bring them when they are in a critical situation as they believe in superstition, so they move around with their patients and sometimes they lose them. Sometimes they come to hospital after they have visited traditional healers but found that their children's illness has become worse.”(IDI-clinical officer)*

*“This ward has many people who live in the remote areas. Once they get ill, they first consult traditional healers since they believe that they have been bewitched. You can just hear “do you know somebody's baby? he is dead!………,he got ill and he was sent to a traditional healer, and when they thought of bringing him to the health facility, he passed away.”(IDI-clinical officer)*

Our findings have also shown that seeking treatment from the traditional healers was sought when there was a persistent fever following treatment from health facilities. If children still had fever after completion of anti-malarial dosage, majority of the participants opted to consult traditional healers for further treatment. Only a few mentioned taking their children back to the health facilities.

*“As we said earlier, in this village, they believe in superstitions. They would say ‘I took my child to the hospital but the fever is still recurring’, then they would think of going to a soothsayer to ascertain the problem.”(FGD-women)*

*“This happens when a child has gone to the hospital for seeking treatment. Having got treatment, they come back home, but if the fever recurs, they think of going to the traditional healers.”(FGD-women)*

This study found that self-medication was commonly practiced by several participants. Many participants reported purchasing drugs from pharmacy/drug shops without prior medical prescription. They explained that they prefer using anti-malarial drugs for the treatment of fever in children. The practice of self-medication was reported by FGD participants from wards located near the health facilities as well as wards which were far off from health facilities. During the interviews with health workers, all of them acknowledged that self-medication was commonly practiced by members of the communities. Heath workers further pointed out that some members of the communities would still visit health facilities if they had found no improvement following self-medication. Health workers explained that the habit of self-medication delays provision of prompt and proper treatment and in most cases results into death. The common reported reasons which influenced many members of the community to opt for self-medication were poor health services from health facilities, shortages of drugs, lack of diagnostic facilities, long distance to the nearby health facility and inability to afford health care charges.

*“It is a long way from the health centre to the patient's residence There are long queues and poor health services because of the shortages of medical staff, therefore the option is to buy Sulphadoxine-pyrimethamine (SP) drug, and if the drug could not help the child, that's when we decide going to the health facility.” (FGD-women)*

*“If the hospital is long distant, you simply buy medicines for the children. But if the situation gets worse, you send them to the health facility.”(FGD-men)*

*“A few individuals bring their children here when they are ill but most parents buy drugs from a pharmacy. There are shortages of drugs at the facility and hence they think there is no alternative way than buying drugs from the pharmacy without a laboratory test.”(IDI-registered nurse)*

### Theme 4: Health workers' practices towards non malaria febrile illnesses

In our study, we found that the diagnosis of febrile patients was mostly done by mRDTs or clinical symptoms/signs as presented by patients with the assistance of Integrated Management of Childhood Illness guidelines. Interviewed health workers explained that mRDTs were used to distinguish malaria from non-malaria fevers and the majority (10/14) prescribed anti-malarial drugs only to patients with mRDT positive result. When patients had negative mRDT result, health workers reported looking for other causes of fever based on clinical signs and history of the fever. Only a few health workers (4/14) stated initiating anti-malarial drugs even if patients had negative mRDT, as one health worker said:

*“…..for instance, we get many patients whom we think do have malaria, but after applying mRDT, the results are different. However, we also offer anti-malarial drugs to those with negative mRDT results and it helps them.” (IDI-clinical officer)*

Moreover, when mRDTs were not available, majority of health workers relied only on clinical manifestations of the patient. When asked to describe symptoms or signs which guide them to make a conclusive diagnosis of febrile illnesses such as malaria, typhoid fever or urinary tract infections, most health workers (12/14) mentioned presence of fever, vomiting, headache, loss of appetite and diarrhea as typical symptoms of malaria. With regards to UTIs, pain during urination was mentioned by majority of health workers as a definitive symptom whereas fewer health workers (2/14) considered urine coloration (from yellowish to milky colour) and small urine volume as symptoms of UTIs. For typhoid fever, symptoms such as abdominal pain and diarrhoea were commonly listed by the majority of health workers.

*“I check symptoms such as fever, or if the parent has told me ‘his child is vomiting’, or has loss of appetite, oh I see! this is definitely malaria and because of these symptoms I initiate anti-malarial treatment. However, even though there is no urine test we think of urinary tract infection as the cause of fever, therefore, we offer anti-malarial drugs and septrin syrup, and this helps!.”(IDI registered nurse)*

It was also revealed that when mRDTs were available many febrile patients tested negative and hence other causes of fever were very likely to be considered. However, in the absence of mRDTs, health workers said several febrile patients were suspected to have malaria and were treated with anti-malarial drugs. They considered that reliance on clinical signs and symptoms only, is prone to lead to misdiagnosis and over-prescription of anti-malarial drugs.

*“Since we got the mRDT last year, I have never found more than eight patients with malaria in a day. However, when MRDTs are out of stock, we prescribe clinical malaria and cure them. Since we started using mRDT, malaria has really decreased though fever is still a big problem. Before mRDT was introduced to us, the situation was even worse.” (IDI-clinical officer)*

Opinions of health workers towards management of persistent fevers following completion of anti-malarial dosage were quite divergent. While majority of health workers (10/14) reported opting for symptomatic diagnosis of non-malaria febrile illnesses and appropriate prescription of antibiotics following failed malaria treatment, however fewer health workers (4/14) reported to switch from first line anti-malarials (artemisinin-based combination therapy) to second line anti-malarial treatment (quinine).

*“If they come back with a persistent fever, we think they could not get cured, so we start the second line therapy such as quinine since initially we gave them Artmether Lumefantrine (ALU).” (IDI-medical attendant)*

### Theme 5: Capacity for diagnosis and management of febrile illnesses in health facilities

Proper management of non-malaria febrile illnesses is largely dependent on the capacity of the health facility to perform accurate diagnosis and treatment of these illnesses. Health workers in this study repeatedly reported lack of diagnostic facilities, shortage of trained health workers, and stock-out of medications as major barriers to proper management of non-malaria febrile illnesses. Our study revealed that from the 12 health facilities, only 2 had diagnostic facilities for a few febrile illnesses; the dispensary had Widal test for detection of typhoid and a microscope used in diagnosis of UTIs and mRDTs, while the health center only had a microscope. The remaining health facilities (10/12) had no diagnostic tests except mRDTs in few health facilities. Health workers explained that they experienced challenges in managing febrile patients without tools for laboratory investigation. Even though mRDT was named as the only diagnostic test which was used to rule out malaria from febrile patients, stock-out of mRDTs was mentioned as an ongoing problem. Health workers said that the supply of mRDTs from the government to the health facilities was normally done on a quarterly basis. However, all health workers repeatedly reported receiving inadequate mRDTs and there were frequent delays in supply of mRDTs. It was clearly stated by health workers that in most cases half way through a quarter, they experienced lack of mRDTs. Among 12 health facilities, only 4 had mRDTs available at the time of our visit. Health workers insisted need for diagnostic tools for malaria and non-malaria febrile illnesses.

*“We don't have a laboratory; so we are trying to exclude other diseases based on symptoms and physical examination. However, knowing that, this is urinary tract infection (UTI) or other infections that can cause fever is difficult; so you simply treat symptomatically.” (IDI-clinical officer)*

*“The first thing we need is a laboratory so that we can know the exact problem and control the misuse of anti-malarial drugs. Also, as I put it earlier, we don't have mRDTs, we simply offer anti-malarials and antibiotics, which is improper use of medicines.” (IDI-clinical officer)*

During the interview with health workers, stock-out of medication was mentioned as a common problem. Drug shortages were reported in most (9/12) health facilities although a few (3/12) had a few boxes of basic drugs such as ALU, antibiotics (amoxicillin, septrin and metronidazole) and pain killer (paracetamol).

*“Shortage of drugs is also a problem. You can place an order to the medical store department but the drugs will not be delivered in time and when delivered, they are usually are insufficient.”(IDI-clinical officer)*

With regards to staffing, our study revealed significant shortage of health workers particularly in health facilities located in rural areas. Among visited health facilities, four (a hospital, two health centers and one dispensary) had more than two health workers at the level of clinical officers. The remaining eight health facilities each had only one clinical officer assisted by a nurse/midwife or medical attendant. Some of the interviewed health workers (medical attendants/nurses) explained that although they prescribe drugs to patients, they had inadequate skills to manage febrile patients. Moreover, health workers reported to work beyond normal working hours and thus attend more patients beyond the standard average number of patients per physician.

*“We have challenge of lack of manpower. We can say, members of staff are there, but in numbers, we are very few. You can be attending a child but a long queue of patients is still waiting for you. Sometimes you can be attending children in their ward; meanwhile other patients are waiting for you at the clinic.” (IDI-registered nurse)*

*“I am alone, but I normally teach my colleagues how to manage patients in my absence. I'm offering training to a nurse midwife and medical attendant. The nurse midwife is able to manage patients but the medical attendant cannot. We do so because of the real situation as you can see we don't have enough health workers, but this doesn't mean that the medical attendant can perform it accurately. The midwife at least can perform better though not at a hundred percent.” (IDI-clinical officer)*

## Discussion

In this study, we report that the awareness of the community on non-malaria febrile illnesses is low. Most of the study participants had little knowledge on non-malaria febrile illnesses and they considered most instances of fever to be synonymous with malaria. Some FGD participants had inappropriate conceptions about the causes of fever. Although the participants reported seeking treatment from health facilities, visiting traditional healers and self-medication were also practiced. Interviewed health workers reported using mRDTs to distinguish non-malaria causes of fever from malaria. However, mRDT negative patients were diagnosed based on symptoms and clinical signs of the patient. In the absence of mRDTs health workers reported giving anti-malarial drugs to many febrile patients as compared to when mRDTs were available. The lack of diagnostic facilities for non-malarial febrile illnesses, stock-out of mRDTs and shortages of drugs were repeatedly reported by all health workers. Therefore, this study revealed inadequate capacity of health facilities to manage febrile illnesses particularly at primary health facilities (dispensaries and health centers).

Fever is a common medical problem in children which may prompt parents to seek medical care. Our results have shown that some participants could not explain the meaning of the term fever and the majority described fever as an illness condition or associated it with symptoms/signs of the disease. In addition, other participants mentioned change of weather and exposure to sunlight as causes of fever in children. The present findings are consistent with the study from Uganda where the study participants used the term fever to describe an illness condition [47] and in Saudi Arabia where more than 70% of parents had a poor understanding of the definition of fever [48]. Knowledge and perception of the community about meaning of fever and its possible causes can influence the decision on seeking treatment from health facility [47]. Additionally, our findings found that the study participants had misconceptions about the causes of fever. Previous studies have also indicated wrong perceptions such as exposure to cold, teething, exposure to sunlight and a warm drink were perceived as causes of fever [49]. Our results indicate the need for correction of such misconceptions within communities as these beliefs would often distract or delay them from seeking treatment from health facilities.

We have indicated minimal awareness of community members on non-malaria febrile illnesses. However, a few participants particularly parents aged more than 30 years were more knowledgeable about non-malaria febrile illnesses as compared to younger parents. Likewise, all interviewed health workers admitted that the communities lack knowledge or had little knowledge on these illnesses. This finding is consistent with results from other studies in Northwestern Ethiopia and Zimbabwe which reported very low awareness of the community regarding non-malaria febrile illnesses [50]–[52]. However, contrary to our findings, previous studies conducted in India, Malaysia and Sri Lanka revealed high level of community awareness on diseases like Chikungunya virus, dengue fever and leptospirosis [53]–[55]. The lack of health education was mentioned by interviewed health workers as a major reason for minimal awareness on non-malaria febrile illnesses. In Tanzania, health education is mainly offered at health facilities [56] however, most people who live in rural areas have limited access to health care facilities. Despite the decrease in malaria incidences, the concept among communities that most fevers are due to malaria was evident. This concept was vividly shown by our results whereas majority of the participants believed that fever is caused by malaria. In addition, interviewed health workers complained that many febrile patients attended health facilities with the assumption that they were suffering from malaria. Our findings correlate with results from other studies in Tanzania, where the term malaria was used to describe fever [57] and any kind of fever was believed to be malarious [58]. This concept is outdated and an obstacle to proper management of patients thus there is a need for creating community awareness on causes of fever other than malaria.

With regards to treatment seeking for febrile illnesses, although some FGD participants reported seeking treatment primarily from health facilities, however, other participants sought treatment from traditional healers. These findings however agree with previous studies in Tanzania where a large percentage of members of the community sought treatment from health care facilities and only a few opted for traditional health care [22], [59]. However, further analysis of health seeking preferences has revealed that those who preferred to seek health care from health facilities also consulted traditional healers since almost half of our study participants reported visiting traditional healers. This observation is in agreement with the report in 2007 from the ministry of Health and Social Welfare (Tanzania), where it was estimated that 60% of those seeking health care services from facilities also depended on traditional healers [60]. Furthermore, although the participants sought health care from health facilities, when symptoms persist they would switch to traditional healers. This finding was shown by the participants who acknowledged going to traditional healers if they were not cured from health facilities. This is also in agreement with a study conducted in Congo, where different treatment options (traditional and health care facilities) were utilized whereby patients initially visited health facilities but when the drugs did not work, they changed to herbs [47]. These findings therefore indicate the need for improving the diagnosis and management of febrile patients at health care facilities so that patients get cured after receiving proper and prompt treatment from health facilities. Alternatively, misdiagnosis and mistreatment of febrile patients at health facilities could continue influencing communities to seek for alternative treatment as revealed by our findings.

Self-medication without medical prescription was found to be a common practice among many FGD participants. Self-medication with anti-malarial drugs has widely been practiced in several countries including Tanzania [22], [61]–[63]. Our findings have also shown this trend, where anti-malarial drugs were self-prescribed by majority of the participants. This behaviour contributes to delay in seeking medical care from health facilities. Also, irrational use of anti-malarial drugs could lead to drug wastage and increased risk of developing parasite resistance [64]. The most commonly reported reasons for self-medication practices were poor health services, shortage of drugs, long distance to the nearby health facility and inability to afford health care charges. In 1994, it was estimated that 72% of Tanzania's population lived within 5 km and 93% within 10 km of health care facilities [65]. However, due to rapid growing population especially in rural areas, the established primary health care system cannot suffice the community demand. At present, the number of villages exceeds the number of health facilities and hence most health facilities serve more than one village [66]. Considering the global decline in malaria prevalence and the reduction in the proportion of fevers due to malaria [11] there is an urgent need to prevent self-medication behavior. Improvement of health service delivery particularly at primary health care facilities will help to reverse this behavior.

The current study has shown that health workers used mRDTs to distinguish non-malaria causes of fever from malaria. However, for the diagnosis of mRDT negative children, health workers reported to rely on symptoms and clinical signs to distinguish non-malaria febrile illnesses. In Tanzania, there has been a roll out of mRDT that was introduced at all levels of health care for parasitological confirmation of malaria prior to treatment [67] as per WHO recommendations [10]. The majority of the interviewed health workers reported giving anti-malarial drugs to only patients with positive mRDT result and looking for other causes of fever when patients had negative mRDT. However, few health workers reported initiating anti-malarial therapy even if the mRDT result was negative. Our findings are supported with results obtained from Kenya whereby 9% of 1,540 patients with negative mRDT were treated with anti-malarials [68]. Several other studies in Tanzania and Zambia have also reported the use of anti-malarials to patients with negative mRDT results [33], [35]. Being dependent on mRDT for the diagnosis of febrile patients, shortage of mRDTs in most health facilities as previously reported from the other study [69] compels health workers to rely on clinical diagnosis and symptoms such as fever, vomiting and loss of appetite to prescribe anti-malarials. However, non-malaria febrile illnesses such as typhoid fever and dengue fever may have similar symptoms with malaria hence are clinically indistinguishable [29], [70]. In addition, health workers reported that when mRDTs were not used many febrile patients were suspected having malaria clinically, however, when mRDTs were applied very few febrile patients were found positive. This indicates that in most cases, health workers put more emphasis on non-malaria illnesses when patients showed negative mRDT or incase there was persistent fever after completion of anti-malaria dosage. Therefore, in the absence of mRDT there is over diagnosis of malaria and under diagnosis of non-malaria febrile illnesses. Clinical diagnosis for febrile illnesses lacks specificity and has been reported to contribute to misdiagnosis and mistreatment of febrile patients [71]. The use of clinical diagnosis which is a normal practice in most health care facilities in the country [72] stands as a major contributing reason for inability to estimate the true prevalence of febrile illnesses in Tanzania [73],[74].

The proper management of non-malarial febrile illnesses depends highly on the availability of diagnostic facilities, professional health workers, medications, transport and communication infrastructures. However, complaints of lack of diagnostic facilities, shortage of health workers, and stock-out of medications were raised by majority of health workers. We found that most health facilities had no diagnostic tests for febrile illnesses. Some non-malaria febrile illnesses are potentially life-threatening and hence failure to diagnose and treat them can result into prolonged and worsening illness with repeated visits to health facilities [75]. Moreover, inappropriate treatment of febrile patients may contribute to a vicious cycle of increasing ill-health and deepening poverty. Therefore, development of rapid diagnostic tests for non-malaria illnesses is of paramount importance [76], [77]. Such tests will be of great importance at primary level of health care where laboratory facilities are scarce. Frequent shortages of drugs were reported to be commonly encountered at health facilities by the majority of interviewed health workers. Likewise, the ongoing problem with drug shortages in Tanzania, was also reported in previous studies [78], [79]. Our findings also have revealed staff shortages in most of the health facilities. Staffing levels and numbers documented in this study are contrary to recommendations by the ministry of health, where a dispensary norm is to have 2 clinicians (clinical officers or assistant medical doctors) and 2 nurses; whereas a health center is intended to be staffed with 4 clinicians and 9 nurses [80]. The challenge of health workforce crisis in Tanzania has been previously reported in other reports as well as by the Tanzanian ministry of health and social welfare [81], [82]. Health workers from our study reported working beyond normal working hours which is also reflected when one considers the ratio of doctor to patients in Tanzania, which stands at 1∶30,000 being far below the standard set by WHO [83]. The issue of staff shortages has led to some health facilities to be operated by unprofessional health workers like medical attendants or nurse/midwife who are not entitled to prescribe drugs other than emergency medication [84]. Similar findings have been found from studies in Tanzania and Uganda where unqualified health workers contributed to inefficiencies in the health service [85] and bypassing of primary health services in rural areas [86].

### Strengths and limitations

FGD participants were selected from different divisions, wards and hamlets, hence they were from diverse demographic backgrounds and their views were a representation of the general population in the district. Interviewed health workers were selected from different health care levels, dispensary to hospital levels. This was purposively done to grasp a wide scope of attitudes and practices by health care staff from the few health facilities which were visited. During focused group discussion with communities more discussions were done with women as compared to men, but this was purposively done because women are responsible for child care in the family hence their views were expected to give a balanced representation of the household health. However, the results of this study showed that both men and women had similar level of knowledge when it comes to perceptions of non-malaria febrile illnesses. It is also possible that some information was lost during the translation of the transcripts before analysis.

### Conclusion

This study has demonstrated that the awareness and level of knowledge of communities on non-malaria febrile illnesses was low. Knowledge from this and other similar studies will provide insights into better and practicable methods for improving the management of febrile patients. The wrong perception among communities, whereas fever is understood as being synonymous with malaria, as encountered in this study pose a challenge to the health sector and thus we emphasizes the need of creating public awareness regarding causes of fever other than malaria. Community misconceptions on fever and its causes must be addressed since such beliefs often distract or delay treatment seeking from health care facilities. It is also crucial that relevant authorities intervene against existing habits of self-medication and seeking treatment from traditional healers. Appropriate treatment of febrile patients will require inputs geared towards strengthening of diagnostic facilities, drugs availability and optimal staffing of health facilities. Therefore, it is advisable that the government and other stakeholders should take appropriate measures to improve health care services delivery.

## Supporting Information

Checklist S1 **STROBE checklist.**
(DOC)

Supporting Information S1 **Topic guide for interviews and discussions.**
(DOC)
