# Supplementary material for: Community Knowledge and Attitudes and Health Workers' Practices regarding Non-malaria Febrile Illnesses in Eastern Tanzania
Source: PLoS Negl Trop Dis. 2014 May 22;8(5):e2896. doi: 10.1371/journal.pntd.0002896 (PMC4031176; doi:10.1371/journal.pntd.0002896)
Supplement: Supporting Information S1 — Topic guide for interviews and discussions. (DOC) [file pntd.0002896.s002.doc]

**TOPIC GUIDE FOR FOCUS GROUP DISCUSSIONS**

Date: _______________________ FGD no: _______________

Name of division:______________ Ward name: _____________

Name of hamlet: ________________ Starting time: _______________

Starting time: _______________ Finishing time: _________________

**SECTION A: KNOWLEDGE ON NON-MALARIA FEBRILE ILLNESSES**

1. What is fever? What causes fever in children? PROBE for explanation of the term fever and its causes.
2. What diseases cause fever in children?
3. Can you mention other illnesses that can cause fever apart from malaria? PROBE for other illnesses including bacterial diseases such as Leptospirosis, Q fever, typhoid fever, respiratory diseases (e.g. Influenza virus), urinary tract infections, relapsing fever, brucellosis, etc and viral diseases such Chikungunya virus, Dengue fever infections, Rotavirus, etc.
4. Which are the common non-malaria febrile illnesses that mostly affect children in this community? For each of the mentioned illness PROBE the mostly affected age group.
5. What do people in this community consider as sources of such illnesses (non-malaria febrile illnesses in children)? PROBE for perceptions on non-malaria febrile illnesses and its causes.

**SECTION B: HEALTH CARE SEEKING BEHAVIOURS**

1. When a child gets fever what do parents or family do? PROBE for health care seeking behaviors; modern biomedical care in health facilities, whether they all come to seek treatment at health facility or if they also seek alternative treatments. If yes which alternative treatments and trace which option is commonly utilized first, followed by which option and reasons for that?
2. If modern biomedical care is not sought at the first place; PROBE the reasons
3. In this community, what do parents do if fever persists in a child after treatment at a health facility? PROBE whether they return the child to the health center or they opt for alternative treatments. Which alternative treatment and the reasons behind it.

**SECTION C: RECOMMENDATIONS**

1. What do you think should be done to reduce non-malaria febrile illnesses? PROBE at different levels; hospitals, health facilities, ministry, government etc.

**TOPIC GUIDE FOR IN-DEPTH INTERVIEWS (IDIS) WITH HEALTH WORKERS**

Date: ______/________/________ Divison name: ____________Ward name:­­­­­­­­­­­­­­­­­­­­­­­­­___________

Harmlet name:______________ Starting time: ____________ Finishing time: __________

**SECTION A: BASIC INFORMATION**

First I will ask you few questions about you and your work.

| **No.** | **Question** | **Answer** |
| --- | --- | --- |
|  | *Indicate health worker gender.* | 1. Male 2. Female |
| 1 | What is your medical qualification?  *(Indicate respondent answer)*  *.* | 1. Specialist doctor (*mention*) ________________________________ 2. General Doctor (Medical Officer) 3. Assistant Medical Officer 4. Clinical Officer 5. Assistant clinical Officer 6. Registered nurse 7. Public health nurse 8. Enrolled nurse 9. Other (*mention*)____________________ |
|  | How long have you been working in this field? (overall experience) | [ ] years |
|  | How long have you been working at this facility as a health care worker? | [ ] years |
|  | On average per week how many patients who come to this health facility present/show symptoms of fever? | [ ] patients per week |

**SECTION B: EXPERIENCE OF HEALTH WORKERS AND AWARENESS OF THE COMMUNITY MEMBERS ON NON-MALARIA FEBRILE ILLNESSES.**

1. Do you think non-malaria febrile illnesses have been affecting people in this community? If YES, PROBE for specific type of illnesses. If NO PROBE reasons for the low prevalence
2. What about incidences of malaria? PROBE for the decrease or increase of malaria cases and the reasons?
3. Do you think the community, particularly parents/guardians who bring their children to this health facility have knowledge (awareness) on non-malaria febrile illnesses? If NO PROBE the reasons for no knowledge or inadequate knowledge.
4. Do people in this community bring their children to health facility when they have fever? Do they bring them when they are ill or very ill? PROBE whether they all come to seek treatment at health facility or if they also seek alternative treatments. If YES which alternative treatments and trace which option is commonly utilized first, followed by which option and reasons for that?

**SECTION C: DIAGNOSIS AND MANAGEMENT OF FEBRILE PATIENTS**

1. What happens if a febrile child is brought to your health care facility? PROBE how they reach final diagnosis and treatment.
2. Which tests do you use for diagnosis of febrile patients? PROBE if they test for malaria and which test do they use (mRDT or microscopy), ask for other laboratory tests they perform and for which diseases.
3. If mRDT/microscopy is not available at health facility and they do clinical diagnosis PROBE what makes him/her think the child has malaria, typhoid fever, urinary tract infection, pneumonia etc. (how do they clinically differentiate febrile illnesses?).
4. If a febrile patient has shown a negative result (following mRDT/microscopy) what happens? PROBE if they do examine for other febrile illnesses? Ask which drugs which are commonly prescribed.
5. Do you think this health facility can manage to offer diagnosis and treatment of non-malaria febrile illnesses? If yes PROBE the available laboratory tests and for which diseases?
6. Does the health facility have adequate infrastructure, staff, equipment, supplies and reliable laboratory testing? PROBE for challenges to manage the illnesses due to the mentioned limitations.
7. Do you think you have adequate skills to manage such diseases? If YES, PROBE for any medical training offered / attended and how often?

**SECTION D: RECOMMENDATIONS ON PROPER MANAGEMNT AND CONTROL OF NON-MALARIA FEBRILE ILLNESSES**

1. What do you think should be done to reduce cases of non-malaria febrile illnesses? PROBE at different levels; at the community and country level?
2. What do you think should be done to improve care and management of non febrile illnesses in public health facilities in the country? PROBE: the role of health facility, district, Government (Ministry of Health) etc.
